# Association between routine and standardized blood pressure measurements and left ventricular hypertrophy among patients on hemodialysis

**DOI:** 10.1186/1471-2369-11-13

**Published:** 2010-06-24

**Authors:** Jaspreet Khangura, Bruce F Culleton, Braden J Manns, Jianguo Zhang, Lianne Barnieh, Michael Walsh, Scott W Klarenbach, Marcello Tonelli,, Magdalena Sarna, Brenda R Hemmelgarn

**Affiliations:** 1 Department of Medicine, University of British Columbia, Vancouver, Canada; 2 Department of Medicine, University of Calgary, Calgary, Canada; 3 Department of Community Health Sciences, University of Calgary, Calgary, Canada; 4 Department of Medicine, University of Alberta, Edmonton, Canada

## Abstract

**Background:**

Left ventricular (LV) hypertrophy is common among patients on hemodialysis. While a relationship between blood pressure (BP) and LV hypertrophy has been established, it is unclear which BP measurement method is the strongest correlate of LV hypertrophy. We sought to determine agreement between various blood pressure measurement methods, as well as identify which method was the strongest correlate of LV hypertrophy among patients on hemodialysis.

**Methods:**

This was a post-hoc analysis of data from a randomized controlled trial. We evaluated the agreement between seven BP measurement methods: standardized measurement at baseline; single pre- and post-dialysis, as well as mean intra-dialytic measurement at baseline; and cumulative pre-, intra- and post-dialysis readings (an average of 12 monthly readings based on a single day per month). Agreement was assessed using Lin's concordance correlation coefficient (CCC) and the Bland Altman method. Association between BP measurement method and LV hypertrophy on baseline cardiac MRI was determined using receiver operating characteristic curves and area under the curve (AUC).

**Results:**

Agreement between BP measurement methods in the 39 patients on hemodialysis varied considerably, from a CCC of 0.35 to 0.94, with overlapping 95% confidence intervals. Pre-dialysis measurements were the weakest predictors of LV hypertrophy while standardized, post- and inter-dialytic measurements had similar and strong (AUC 0.79 to 0.80) predictive power for LV hypertrophy.

**Conclusions:**

A single standardized BP has strong predictive power for LV hypertrophy and performs just as well as more resource intensive cumulative measurements, whereas pre-dialysis blood pressure measurements have the weakest predictive power for LV hypertrophy. Current guidelines, which recommend using pre-dialysis measurements, should be revisited to confirm these results.

## Background

Hypertension is common among patients on hemodialysis (HD) and is associated with an increased risk of coronary artery disease, congestive heart failure, cerebrovascular complications, mortality and left-ventricular (LV) hypertrophy[1-3]. Cardiovascular disease accounts for the majority of deaths in patients with end stage renal disease (ESRD)[4,5], thus adequate blood pressure (BP) control is important to reduce the risk of adverse cardiac events. LV hypertrophy, in itself, affects up to 80% of ESRD patients[2], is an established cardiac manifestation of chronic hypertension[6] and an independent predictor of cardiovascular events and mortality in both the general and ESRD population[7,8]. Although the relationship between hypertension and cardiovascular morbidity and mortality is not consistently reported in the HD patient population[9], it is generally accepted that BP control is important for cardiovascular risk reduction in these patients[10]. Recommendations for BP measurement among patients on dialysis, including timing (pre- vs. post-dialysis), target levels, and methods of measurement, are variable, while in general, blood pressure control in HD patients remains poor[1,11].

Although the most accurate BP measurement technique among patients on HD is unknown, it has been demonstrated that casual dialysis-unit BP measurements differ considerably when compared to ambulatory and standardized BP measurements[12-14], and correlate poorly with target organ damage[13]. Further, it was shown that BP measurements obtained outside the dialysis unit (home and ambulatory BP measurements) were more strongly correlated with LV hypertrophy than those obtained within the dialysis unit[12,13]. Previous studies have produced inconsistent results with respect to the BP measurement method which best predicts LV hypertrophy[15,16].

By using more sensitive measures of LV mass, and more frequent measurements of BP, we sought to determine the agreement between various BP measurement methods, at different times (pre-, intra- and post- dialysis) among patients on HD. We also sought to determine the association between BP measurement methods and LV hypertrophy. Further, we hypothesized that BP values obtained by a standardized BP protocol (as recommended by national guidelines) would be more closely associated with LV hypertrophy than BP values obtained by other techniques.

## Methods

### Subjects

This study involves a post-hoc analysis of data collected within a previously reported randomized controlled trial, where details of the subjects and protocol are previously described[17]. Subjects included in this analysis were limited to those from the University of Calgary who underwent cardiac magnetic resonance imaging (cMR) as a baseline procedure during the clinical trial, but prior to the intervention. Subjects from the University of Alberta were excluded because of the absence of serial dialysis-associated BP measurements.

Eligible subjects were 18 years of age or older, receiving in-center, self-care or home hemodialysis three times a week. The study protocol was approved by the University of Calgary bioethics committee.

### Standardized BP at baseline

Standardized BP measurements were obtained at baseline by a physician using a mercury sphygmomanometer in accordance with the Canadian Hypertension Education Programs protocol for blood pressure measurement[18]. Three sitting BP measurements were taken 5 minutes apart, after the patient had been resting in a quiet room for at least 5 minutes. The average of the last two measurements was utilized as the standardized BP value for this study[17]. The standardized BP measurements were obtained prior to dialysis but within two weeks of the cMR exam.

### Casual BP measurements at a single hemodialysis session at baseline

A single pre- and post dialysis BP measurement was taken by a dialysis unit nurse with patients in a sitting position, within 30 minutes prior to and following the dialysis session using either the automated GAMBRO Phoenix or manually using a mercury sphygmometer on the non-fistula arm. Intra-dialytic BPs were recorded every 15-30 minutes during dialysis. The average value of all of the BP measures taken during a single dialysis session, which ranged from 2 to 11 readings per dialysis session (median 7), was used to determine the intra-dialysis BP.

### Cumulative BP measurements

Monthly pre-, intra- and post-dialysis BP measurements were retrospectively collected from hemodialysis records for a single hemodialysis session each month during the 12 month period prior to the cMR examination. These casual BP measurements were obtained by dialysis staff or by the patient (for those patients on home conventional hemodialysis).

### Left ventricular mass and left ventricular hypertrophy

cMR was performed on 1.5-T MRI systems (Avanto^® ^or Sonata^®^; Siemens Medical Solutions, Erlangen, Germany) with 8-channel cardiac coils. Standard, breath-held, retrospectively electrocardiogram-gated gradient-echo sequences (steady state free precession, SSFP) in contiguous short-axis views (25 phases, slice thickness 8 mm) were applied. The evaluation of the cMR images was performed in a professional core lab (CIRCLE International Ltd., Calgary, Canada) by readers blinded to BP measurement data. Papillary muscles were included in the LV myocardial mass calculation. The formula by DuBois and DuBois[19] was used to index LV mass to body surface area. The presence of LV hypertrophy was defined as LV mass/m^2 ^>83 for males and LV mass/m^2 ^>67 for females[20].

### Laboratory measurements

Lab records for the 12 months prior to the cMR procedure were extracted from the Southern Alberta Renal Program database. Given the small sample size, we chose, a priori, to limit our assessment to serum calcium, phosphate, hemoglobin and parathyroid hormone (PTH) as these have been linked to alterations in LV structure[21]. Serum calcium was not corrected for serum albumin values. PTH was measured using the Nichols bio-intact assay. Mean values were collected at baseline and in the 12 months prior to the cMR.

### Statistical analysis

Subject characteristics are presented as mean ± standard deviation (SD) or median and inter-quartile range (IQR) for continuous variables as appropriate, and number (percent) for categorical data. All BP measurements are reported as mean ± SD. Agreement between BP measurement methods was assessed using two statistical methods. Lin's[22,23] concordance correlation coefficient (CCC) was used to measure overall agreement between any two measurement methods. Lin's CCC incorporates both bias and precision, with a CCC value of 1 indicating perfect agreement and -1 indicating perfect inverse agreement. CCC values are reported with their respective 95% confidence intervals (CI). The second statistical method used to assess agreement was the Bland and Altman method[24], which plots the difference between pairs of measurements on the y-axis against the mean of each pair on the x-axis. The 95% CI are also presented. Pearson's product moment correlation coefficients are reported for each method against LV mass, standardized to body surface area.

Multivariate linear regression analyses, with LV mass corrected for body surface area as the dependent variable and BP parameters as independent variables, were used to determine the potential confounding effects of hemoglobin, calcium-phosphate product, PTH, age and dialysis vintage. Receiver operated characteristic (ROC) curves, and area under the curve (AUC) statistics, with their respective 95% CI, were generated to compare the test performances of various BP methods in detecting the presence of LV hypertrophy. Only the results for systolic blood pressure (SBP) are shown as the results of the diastolic blood pressure analysis did not appreciably change the outcomes of this study. All analyses were performed using Stata 9.2 (Stata Corporation, College Station, Texas).

## Results

### Patient and baseline characteristics

**Figure 1:**
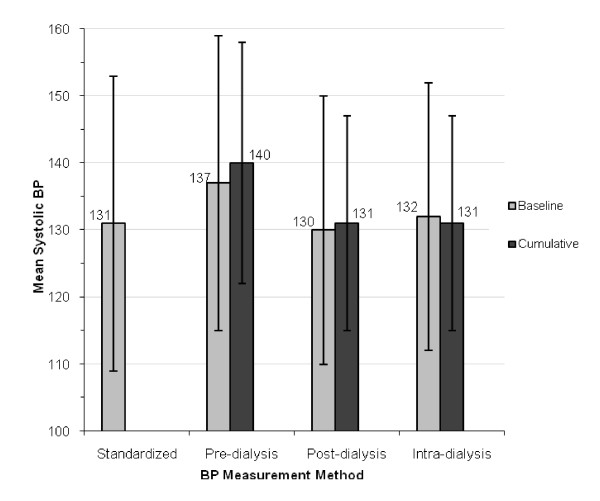
**Summary of systolic blood pressure measurements by different methods (n = 39) **.

A total of 39 patients were included in the study. The mean age of study subjects was 54 years and two-thirds were male (Table 1). The majority of subjects were taking antihypertensive medications, the most common being angiotensin converting enzyme (ACE) inhibitors or angiotensin receptor blockers. LV hypertrophy, as measured by cMR, existed in 29 (74.4%) study subjects. Blood pressure results obtained by the various methods are detailed in Figure 1. Pre-dialysis BP values were higher than post-dialysis BP values, and both baseline and cumulative pre-dialysis BPs were higher than the standardized BP taken pre dialysis.

**Table 1:** Baseline characteristics of study subjects

**Characteristic **^**†**^	N = 39
Age, years	54.4 ± 13.3
Male gender, n (%)	26 (66.6)
Caucasian, n (%)	33 (84.6)
Body mass index (kg/m^2^)	25.3 ± 5.4
Time on dialysis, years	5.0 ± 4.6
Prior renal transplantation, n (%)	12 (30.8)

Cause of ESRD, n (%)	
Diabetic nephropathy	12 (30.0)
Hypertension/vascular	3 (7.7)
Glomerulonephritis	10 (25.6)
Polycystic kidney disease	4 (10.3)
Urologic	4 (10.3)
Other	6 (15.4)

Comorbid illnesses, n (%)	
Ischemic heart disease	17 (43.6)
Congestive heart failure	10 (25.6)
Peripheral vascular disease	6 (15.4)
Cerebrovascular disease	6 (15.4)
Diabetes mellitus	16 (41.0)

Medication use, n (%)	
ASA	17 (54)
ACE inhibitor or ATII antagonist	25 (64)
Calcium channel blocker	21 (54)
Beta-blocker	16 (41)
Other anti-hypertensive	5 (13)
Any anti-hypertensive	33 (85)

Hemoglobin, g/dL	121 ± 13.7
Serum calcium, mmol/L	2.32 ± 0.24
Serum phosphate, mmol/L	1.69 ± 0.43
Calcium-phosphate product, mmol^2^/:L^2^	3.94 ± 1.10
Parathyroid hormone, pg/ml	239 (83, 391)
Left ventricular mass	173.7 ± 61.0
Left ventricular mass/m^2^	93.5 ± 31.6
Left ventricular hypertrophy, n (%)	29 (74.4%)

^†^Reported as mean ± SD or median (IQR) where data is skewed for continuous variables and number (%) for categorical data.

### Agreement between BP measurement methods

The agreement levels for the different methods for SBP measurements are shown in Table 2. Agreement between methods was highly variable, from a CCC of 0.35 (95% CI 0.05, 0.59) for baseline pre-dialysis and standardized methods to a CCC of 0.94 (95% CI 0.89, 0.97) for cumulative intra-dialysis and cumulative post-dialysis methods. The 95% CI for the CCC's for all methods overlapped, suggesting no significant difference in agreement between BP measurement methods. The Bland-Altman analysis confirmed this, showing no consistent bias between the different methods.

**Table 2:** Agreement levels between different methods for all systolic blood pressure measurements.

		*Baseline measurements*				*Cumulative measurements*		
	
		*Standard*	*Pre-dialysis*	*Post-dialysis*	*Intra-dialysis*	*Pre-dialysis*	*Post-dialysis*	*Intra-dialysis*
	***Standardized***							

		*-5.8 (-13.9, 2.2)*						
*Baseline*	*Pre- dialysis*	*(-54.5, 42.9)*	---	---	---	---	---	---
		*0.35 (0.05, 0.59)*						
	
		*1.0 (-5.3, 7.3)*	*6.8 (0.4, 13.2)*					
	*Post -dialysis*	*(-37.0, 39.0)*	*(-31.9, 45.5)*	---	---	---	---	---
		*0.58 (0.33, 0.75)*	*0.52 (0.27, 0.71)*					
	
		*-1.1 (-7.0, 4.7)*	*4.6 (-1.8, 11.0)*	*-2.2 (-5.5, 1.0)*				
	*Intra-dialysis*	*(-37.6, 35.1)*	*(-34.0, 43.2)*	*(-21.8, 17.3)*	***---***	***---***	***---***	***---***
		*0.62 (0.39, 0.78)*	*0.55 (0.29, 0.73)*	*0.87 (0.77, 0.93)*				

		*-8.6 (-14.3, -2.8)*	*-2.7 (-8.4, 2.9)*	*-9.6 (-15.0, -4.1)*	*-7.3 (-12.1, -2.5)*			
*Cumulative*	*Pre- dialysis*	*(-43.2, 26.1)*	*(-36.7, 31.2)*	*(-42.5, 23.3)*	*(-36.4, 21.8)*	***---***	***---***	***---***
		*0.57 (0.34, 0.73)*	*0.62 (0.39, 0.78)*	*0.54 (0.31, 0.71)*	*0.65 (0.45, 0.79)*			
	
		*0.6 (-4.5, 5.8)*	*6.5 (0.2, 12.8)*	*-0.36 (-5.1, 4.4)*	*1.9 (-2.6, 6,3)*	*9.2 (5.5, 12.9)*		
	*Post-dialysis*	*(-30.4, 31.7)*	*(-31.60 44.5)*	*(-29.1, 28.4)*	*(-25.1, 28.8)*	*(-13.2, 31.6)*	---	---
		*0.67 (0.47, 0.80)*	*0.46 (0.20, 0.66)*	*0.67 (0.47, 0.81)*	*0.72 (0.54, 0.84)*	*0.67 (0.49, 0.80)*		
	
		*0.2 (-5.0, 5.4)*	*6.0 (0.0, 12.0)*	*-0.8 (-5.4, 3.8)*	*1.4 (-2.4, 5.3)*	*8.8 (5.8, 11.7)*	*-0.4 (-2.3, 1.3)*	
	*Intra-dialysis*	*(-31.2, 31.6)*	*(-30.4, 42.4)*	*(-28.5, 27.0)*	*(-21.9, 24.8)*	*(-9.1, 26.6)*	*(-11.4, 10.5)*	---
		*0.65 (0.46, 0.79)*	*0.49 (0.25, 0.68)*	*0.69 (0.50, 0.82)*	*0.78 (0.64, 0.87)*	*0.75 (0.61, 0.85)*	*0.94 (0.89, 0.97)*	

Each cell contains the mean difference (95% confidence interval) (first row), 95% agreement limits (second row) and CCC (95% confidence interval) (third row).

^† ^Mean difference was calculated by subtracting the row from the column.

Although not statistically significant, standardized, post- and intra-dialysis measurements (baseline and cumulative) had moderate to high agreement (CCCs 0.67 to 0.94), while the comparison of each method with baseline pre-dialysis BPs showed lower levels of agreement (CCCs 0.35 to 0.62).

### Association between BP measurement method and LV mass

Correlations between BP measurement methods and LV mass are shown in Table 3. Standardized BP had a correlation of r = 0.44 (P = 0.005). Baseline post- and intra-dialysis methods had correlations of 0.60 (P = 0.0001) and 0.59 (P < 0.0001) respectively, slightly higher than the correlation for cumulative post- and intra- dialysis methods. The correlation between BP method and LV mass was significant (P < 0.05) for all methods except baseline pre-dialysis (r = 0.30, P = 0.068). Results were unchanged when adjusted for hemoglobin, calcium-phosphate product, PTH, age and dialysis vintage.

**Table 3:** Pearson's product correlation coefficients for baseline and cumulative systolic BP measurement methods with LVM/m^2 † ^and ROC statistics for LV hypertrophy^††^

BP Measurement Method		**Correlation with LVM/m **^**2**^		ROC AUC (95% Confidence Interval)
		Pearson's r P value		
	Standardized	0.44	0.005	0.79 (0.64, 0.94)

	Pre-dialysis	0.30	0.068	0.71 (0.53, 0.89
Baseline	Post-dialysis	0.60	0.0001	0.79 (0.64, 0.93)
	Intra-dialysis	0.59	<0.0001	0.80 (0.64, 0.96)

	Pre-dialysis	0.38	<0.0001	0.78 (0.62, 0.95)
Cumulative	Post-dialysis	0.53	<0.0001	0.74 (0.57, 0.92)
	Intra-dialysis	0.51	0.0009	0.74 (0.58, 0.90)

^† ^LVM/m^2 ^= LVM indexed to patient's BSA, using the formula described by DuBois and DuBois^19^

^†† ^LV hypertrophy was defined as an LVM/m^2 ^>83 for males and LVM/m^2 ^>67 for females^20^

**Figure 2:**
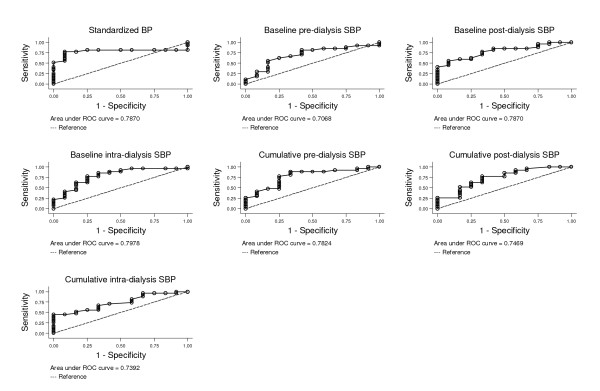
**ROC curves for different systolic BP measurement methods **. The diagonal dotted line indicates a hypothetical test with no predictive value

ROC curves for the SBP measurements and LV hypertrophy are shown in Figure 2 and their respective AUC values are provided in Table 3. All the SBP values were related to LV hypertrophy. All of the BP measurement methods had similar AUC values and their 95% CIs overlapped; hence they were not significantly different from each other. AUC values were highest for standardized systolic (AUC 0.79), baseline post- (AUC 0.79) and baseline intra-dialysis (AUC 0.80). Both cumulative intra- and post-dialysis methods had an AUC of 0.74 while the AUC for the baseline pre-dialysis method was 0.71.

## Discussion

In this study we investigated the agreement between standardized and casual (single dialysis session and 12 month cumulative) BP measurement methods, and their association with left ventricular mass in hemodialysis patients. Current guidelines [25,26] recommend the use of a single pre-dialysis BP measurement for the care and management of chronic hemodialysis patients. However our analysis of pre-, intra-, post- and standardized BP measurements suggests that a single pre-dialysis measurement is the least accurate method of BP assessment and demonstrates the weakest performance at predicting LV hypertrophy compared to cumulative and standardized measurements.

Intra- or post- dialysis BP measurements collected over several months may provide a more accurate assessment of BP in a stable, chronic HD patient rather than a single pre-dialysis measurement. However, this is resource intensive and often impractical in the clinical setting. Furthermore, in spite of extremely high agreement (CCC of 0.94), cumulative intra- and post-dialysis BP measurements were not the strongest predictors of LV hypertrophy, which is the more clinically significant outcome. BP values obtained by the standardized BP measurement protocol were closely associated with LV mass, but no more so than a single intra-dialysis or single post-dialysis measurement, all three of which had similar and strong predictive power for determining LV hypertrophy.

While the relationship between BP and mortality in ESRD patients is not clear[9,27,28], it is well accepted that high BP contributes to LV hypertrophy - which itself is an independent risk factor for morbidity and mortality in the ESRD population[7,8]. The prevalence of LV hypertrophy in our study, 74.4%, is similar to that reported in prior studies[13,29].

Two previous studies of BP as a predictor of LV geometry both found pre-dialysis BP's to be strong determinants of LV hypertrophy[15,16]. These studies however were small, used a less sensitive measure of LV mass (echocardiography), and only measured BP over 12 dialysis sessions. A more recent study of 140 hemodialysis patients reported that out-of-clinic recordings (1-week-averaged home BP readings) performed better at predicting LV hypertrophy than routine and standardized BP recordings obtained pre- and post-dialysis in the dialysis unit[13]. Unfortunately, access to home BP monitors or supervised ambulatory BP monitoring is not always available and convincing patients to measure BP at home when it is measured so frequently in HD units, may be difficult.

BP management in HD patients is partly dependent on the ability of physicians to accurately assess BP levels, which remains a challenge. In the absence of a universally accepted method for BP assessment among patients on hemodialysis, we selected standardized BP as the gold standard for the purpose of this study, as it was based on a protocol established and endorsed by national hypertension guidelines[18]. Previous studies provide conflicting results as to whether pre-dialysis, post-dialysis, inter-dialytic or a combination of BP measurements methods are most valuable for clinical decision making[15,30,31]. However the issue of accuracy of BP measurement in the dialysis unit is often ignored. Our results are consistent with previous reports [11-14,32] suggesting that routine dialysis BP measurements are in fact highly inaccurate.

Our results should be interpreted in the context of the study limitations. Firstly, our sample size was small, including 39 patients. Despite the small sample size, our use of cMR enabled us to use a more accurate measure of LV mass; thus our point estimates are more precise than those of previous studies[13]. Secondly, as a single-centre observational study, the generalizability of our results could be questioned. However the lenient inclusion and exclusion criteria does increase generalizability of the results. The cross-sectional nature of the data for baseline BP measurement, lack of ambulatory blood pressure measurements and assessment of LV hypertrophy without an assessment of volume status prevents us from drawing firm conclusions, however, the results are further supported by cumulative BP measurements obtained over a 12 month period. Finally, our study included patients with a wide range of ESRD etiologies, including diabetes and heart failure, both of which contribute to LV hypertrophy. The autonomic and cardiovascular dysfunction in these disease processes could have confounded the BP-LV hypertrophy correlation. Further studies are necessary to delineate whether these co-morbidities affect the BP-LV hypertrophy relationship.

## Conclusion

In summary, we found that a single standardized BP measurement has strong predictive power for LV hypertrophy, but is not significantly better than casual post- and intra-dialysis BP measurements taken at a single point in time or over several months. For ease of practice, a single standardized measurement performs just as well as the more resource intensive cumulative measurements. Given the poor agreement with other methods, and relatively poor predictive power of a single pre-dialysis measurement in predicting LV hypertrophy, the results of this study also suggest that the current practice guidelines, which advocate the use of pre-dialysis measurements for chronic hemodialysis patients, warrants further study. This study does not however provide strong evidence to disregard current guidelines.

## Abbreviations

HD: hemodialysis; LV: left ventricular; ESRD: end stage renal disease; BP: blood pressure; cMR: cardiac MRI; PTH: parathyroid hormone; SD: standard deviation; IQR: inter-quartile range; CCC: concordance correlation coefficient; ROC: receiver operated characteristic; AUC: area under the curve; CI: confidence intervals; SBP: systolic blood pressure; ACE: angiotensin converting enzyme

## Competing interests

The authors declare that they have no competing interests.

## Authors' contributions

BRH, MT, SWK, BJM, and BFC made substantial contributions to conception, design and acquisition of data and funds. JP, BRH, JZ, BJM and BFC made substantial contributions to conception and design. All authors made substantial contributions to interpretation of the data, all authors were involved in revising the manuscript for important intellectual content, and all authors have read and approved the final manuscript.

## Pre-publication history

The pre-publication history for this paper can be accessed here:

http://www.biomedcentral.com/1471-2369/11/13/prepub
